# Immune-mediated diseases are associated with a higher risk of ALS incidence: a prospective cohort study from the UK Biobank

**DOI:** 10.3389/fimmu.2024.1356132

**Published:** 2024-03-05

**Authors:** Wen Cao, Zhi Cao, Lu Tang, Chenjie Xu, Dongsheng Fan

**Affiliations:** ^1^ Department of Neurology, Peking University Third Hospital, Beijing, China; ^2^ Beijing Key Laboratory of Biomarker and Translational Research in Neurodegenerative Disorders, Beijing, China; ^3^ School of Public Health, Zhejiang University School of Medicine, Hangzhou, China; ^4^ School of Public Health, Hangzhou Normal University, Hangzhou, China

**Keywords:** amyotrophic lateral sclerosis, IMD, UK Biobank, immunity, prospective cohort

## Abstract

**Objective:**

The occurrence of immune-mediated diseases (IMDs) in amyotrophic lateral sclerosis (ALS) patients is widely reported. However, whether IMDs and ALS is a simple coexistence or if there exists causal relationships between the two has been a subject of great interest to researchers.

**Methods:**

A total of 454,444 participants from the prospective cohort of UK Biobank were recruited to investigate the longitudinal association between IMDs and ALS. Previously any IMDs and organ specific IMDs were analyzed in relation to the following incident ALS by Cox-proportional hazard models. Subgroup analyses were performed to explore the covariates of these relationships.

**Results:**

After adjusting for potential covariates, the multivariate analysis showed that any IMDs were associated with an increased risk of ALS incidence (HR:1.42, 95%CI:1.03-1.94). IMDs of the endocrine-system and the intestinal-system were associated with increased risk of ALS incidence (endocrine-system IMDs: HR:3.01, 95%CI:1.49-6.06; intestinal system IMDs: HR:2.07, 95%CI: 1.14-3.77). Subgroup analyses revealed that immune burden, including IMD duration and the severity of inflammation had specific effects on the IMD-ALS association. In participants with IMD duration≥10 years or CRP≥1.3mg/L or females, previous IMDs increased the risk of incident ALS; however, in participants with IMD duration <10 years or CRP<1.3mg/L or males, IMDs had no effect on incident ALS.

**Interpretation:**

Our study provides evidence that previous any IMDs and endocrine-system and the intestinal-system specific IMDs are associated with an increased risk of developing ALS in females, but not in males.

## 1 Introduction

Amyotrophic lateral sclerosis(ALS) is one of the most rapidly progressing and fatal neurodegenerative diseases, involving degeneration and death of upper and lower motor neurons. The median survival period after the onset of symptoms is 3-5 years (1). The exact aetiology of ALS remains not well defined, but there is increasing evidence suggesting the potential involvement of dysregulated immune response.

The precise contribution of the immune system in ALS pathogenesis is an active area of research. Currently, compelling evidence implicates that aberrant immune changes are not only significant features of ALS, but may be risk factors of ALS onset: 1) Our previous findings have demonstrated that higher neutrophils and its derived ratios neutrophil-to-lymphocyte ratio (NLR) and systemic immune-inflammation index (SII) are associated with higher risk of ALS incidence (2, 3); 2) Gene mutations in ALS involve inflammatory pathways. For instance, the most common genetic cause of ALS, the intermediate allele of C9orf72, is believed to be associated with systemic autoimmune diseases (4). Loss-of-function of C9orf72 lead to fatal autoimmune diseases in mice, indicating the role of C9orf72 in immune regulation (5). Recently discovered ALS mutation TBK1 is a major regulator of innate immune system signaling and plays a significant role in autophagy and inflammation (6); 3) Evidence from mendelian randomization revealed a causal link between peripheral immune abnormalities and incident ALS (7).

Immune-mediated diseases (IMDs) are a group of common chronic diseases, with an estimated prevalence ranging from 5-10% (8). The main pathogenesis of IMDs involves the mistake attack of one’s own tissues by the immune system. The relationship between IMD and ALS is complex: 1) The occurrence of IMDs in ALS patients is widely reported, and patients with coexisting ALS and IMDs had shorter survival latency than those with pure ALS (9); 2) The immune dysregulation observed in ALS patients, including abnormalities in innate immune system (activation of central microglial cells, increased peripheral NK cells and neutrophils, peripheral monocyte infiltration into the central nervous system, elevated levels of inflammatory factors, and alterations in gut microbiota (10–13), and adaptive immune system (changes in T cells) (14, 15), are also significant features of IMDs (8, 16–18); 3) Existing research has revealed common genetic overlap structures between IMDs and ALS (19). The above findings support the idea that IMDs and ALS share similar genetic, molecular, and cellular pathways, which may predict the onset of ALS. However, whether IMDs and ALS is a simple coexistence or if there exists causal relationships between the two has not been clarified.

To date, only two retrospective cohorts have focused on the relationships between IMDs and ALS (20, 21). Due to the low incidence of ALS, no large-scale prospective studies have explored the relationship of IMDs and ALS. Moreover, few studies have taken into account the duration of IMDs and the severity of inflammation, which are two significant parameters reflecting the burden of immune dysregulation (22).

To address this significant research gap, we utilized a prospective multicenter cohort of the UK Biobank to investigate the association between previous IMDs and ALS. Firstly, we analyzed the relationship between any IMD and organ-specific IMDs with the risk of ALS incidence. Secondly, we examined whether an extended duration of IMDs and an increased severity of inflammation further elevated the risk of ALS.

## 2 Methods

### 2.1 Study population

UK Biobank is a population-based prospective cohort study that aimed to investigate the genetic, lifestyle, and environmental causes of diseases. Between 2006 and 2010, more than 500,000 subjects aged 40-70 years were recruited at 22 assessment centers across the UK (https://www.ukbiobank.ac.uk/). All participants were registered with the UK National Health Service. At the recruitment visit, participants completed a self-administered touchscreen questionnaire on sociodemographic characteristics, lifestyle exposures, medical history, medication use and underwent physical measurements.

All participants provided written informed consent. All data were deidentified. The UKB received ethical approval from the UK National Health Service, National Research Ethics Service North West, the National Information Governance Board for Health and Social Care in England and Wales, and the Community Health Index Advisory Group in Scotland. In addition, an independent ethics and governance council was formed to oversee its continued adherence to the ethics and governance framework. The current study was approved by the UK Biobank (application number 79095). This study followed the Strengthening the Reporting of Observational Studies in Epidemiology (STROBE) reporting guideline.

### 2.2 Ascertainment of ALS

ALS cases were identified through a comprehensive review of medical records, comprising inpatient health records obtained from hospitals across England (89%), Scotland (7%), and Wales (4%) using the Hospital Episode Statistics Admitted Patient Care (HES APC) system in England, Scottish Morbidity Records (SMR01) in Scotland, and Patient Episode Data for Wales (PEDW). The date of initial ALS diagnosis was determined as the first available date of ALS confirmation. Dates of death were determined by cross-referencing with the national death registry data.

The International Classification of Diseases 10 revision (ICD10) codes were used to record disease diagnoses.The outcome of interest was ALS incidence. Incident ALS cases within the UK Biobank were identified by ICD-10 codes through linkage registry (G12.2). In the current study, we excluded participants who had previous ALS at baseline (n=35).

### 2.3 Ascertainment of immune-mediated diseases

As suggested in previous studies (23, 24), a range of 41 IMDs were identified by matching the ICD-10 codes from hospital inpatient data. The diagnosis date was the earliest date recorded in participants inpatient register. The longitudinal associations of any IMDs and the organ specific IMDs were assessed with incident ALS. For organ specific IMDs, we assessed 8 organ-specific diseases for their associations with ALS. To ensure the IMDs were diagnosed before ALS onset and avoid the reversed association, we required the immune-mediated disease diagnosis to be present before the baseline recruitment.

### 2.4 Baseline characteristics

Smoking and drinking status were collected using touch-screen questionnaire. Height and weight were measured directly and body mass index was calculated using the usual formulae (weight, kg/height^2^, m^2^). The Townsend deprivation index (TDI), widely used as an indicator of neighborhood socioeconomic circumstances and based on study member residential address, is continuously scored with higher values denoting greater deprivation. The highest qualification of participants were achieved via touch-screen questionnaire.

405393 participants were measured for high sensitivity C-reactive Protein (CRP) in our analysis. Serum high sensitivity CRP levels were analyzed using the Beckman Coulter AU5800 apparatus via a standard method of immunoturbidimetry.

### 2.5 Statistical analysis

All individuals were followed up from the date of recruitment until that of ALS diagnosis, death, or the end of the study period (September 16, 2021), whichever occurred first. Individuals with prevalent IMDs reported at baseline enrollment were classified as the exposure group. Individuals with either prevalent IMDs reported after baseline enrollment or incident ALS reported before baseline were removed.

Time-to-event analyses was used to estimate the hazard ratio (HR) and 95% confidence interval (CI) by Cox regression models. Model 1 was adjusted for demographic variables including age at recruitment and sex. Model 2 was further adjusted for socioeconomics factors, including education and Townsend deprivation index(TDI). Model 3 were further adjusted for lifestyle factors (smoking status, drinking, BMI).

We performed a series of sensitive analyses to test the robustness of our results. To evaluate the potential reverse causation biases, we performed the sensitivity analyses by excluding the ALS patients with the less than 1 year and 3 years.

In stratification analyses, we examined the association between different inflammation loads using the median of CRP which was measured at baseline and different IMD duration (<5 years, 5-10 years and >10 years). As a biomarker for the severity of inflammation, CRP is associated with a high risk of ALS (25, 26). The duration of IMDs may signify a prolonged period of low-grade inflammation, possibly reflecting the long-term physical state that previous research has not fully elucidated. As IMD patients were more likely to be females (27), we also examined the sex effect on IMD-ALS association.

All the above statistical analyses were performed with R (version 4.2.2; R Foundation for Statistical Computing, Vienna, Austria) and R packages (“survival” and “survminer” packages).

## 3 Results

### 3.1 Baseline characteristics

**Figure 1:**
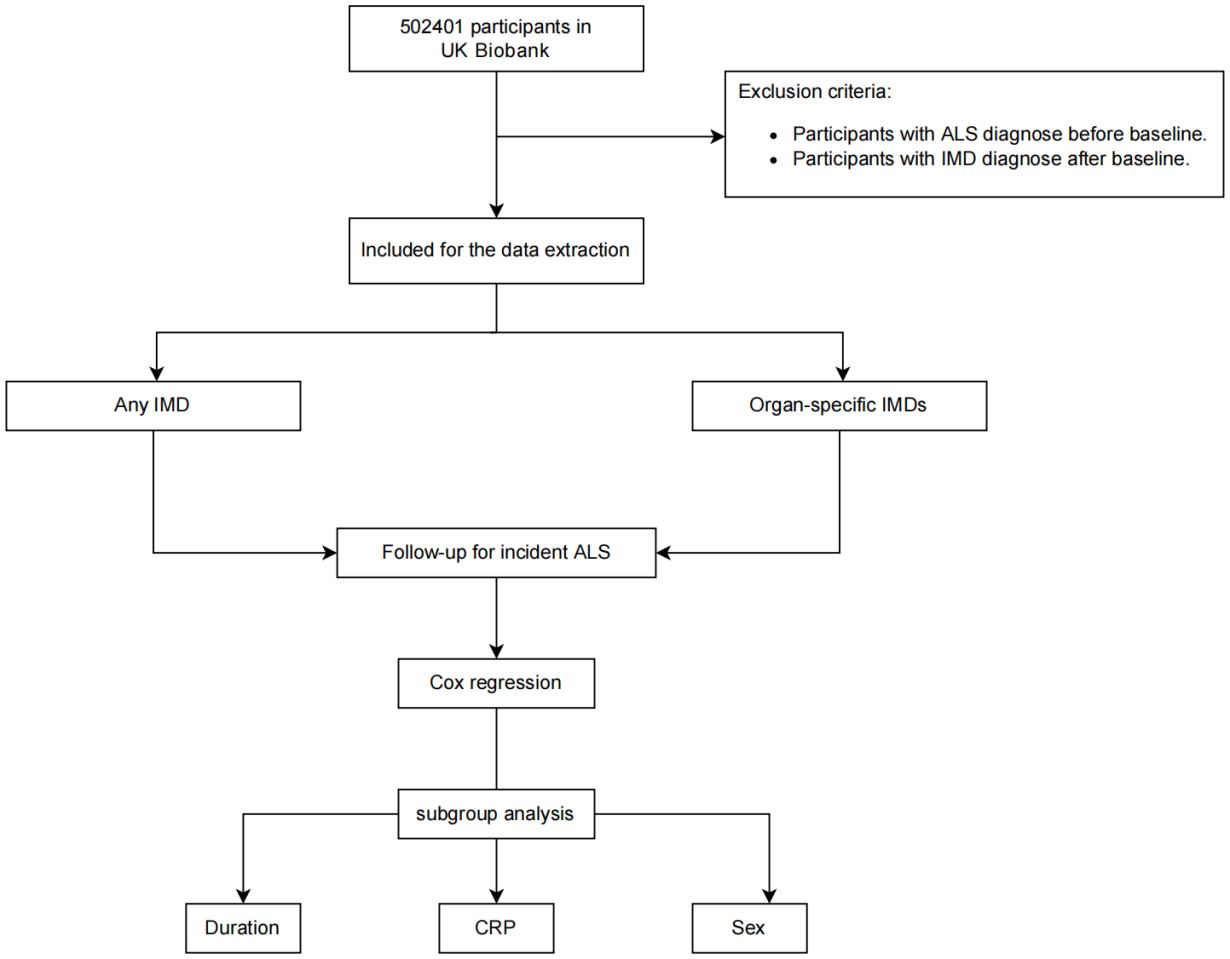
Flowchart of the study. Participants with ALS at baseline and with IMDs after baseline were removed for the Cox proportional hazards analysis.

The baseline characteristics were shown in **Table 1**. A total of 434,444 participants were recruited to the following analyses. The mean age was 56.27 years old. Of all, 45.8% were males (199,018/434,444). 29,813 participants had at least 1 immune-mediated disease. Compared with participants without any IMDs, participants with IMDs were more likely to be females and older in age. Moreover, participants with IMDs were more likely to have higher BMI, higher smoking frequency, higher TDI. They were less likely to have a college or university education and to consume alcohol (**Table 1**). Notably, more participants with IMDs (0.2%) developed ALS than non-IMD participants (0.1%) The flowchat of our study was shown in **Figure 1**.

**Table 1:** Baseline characteristics of participants with or without IMDs in UKB.

	Without IMDs	With IMDs	P-value
	(N=404631)	(N=29813)	
Sex			
Male	186959 (46.2%)	12059 (40.4%)	<0.001
Female	217672 (53.8%)	17754 (59.6%)	
Age (years)			
Median [Q3-Q1]	57.0 [63.0-50.0]	59.0 [64.0-52.0]	<0.001
BMI (kg/m2)			
Median [Q3-Q1]	26.6 [29.6-24.0]	27.7 [31.6-24.7]	<0.001
Smoking			
Never	224522 (55.5%)	14688 (49.3%)	<0.001
Previous	135799 (33.6%)	11466 (38.5%)	
Current	42090 (10.4%)	3406 (11.4%)	
TDI			
Median [Q3-Q1]	-2.19 [0.419–3.67]	-1.57 [1.56–3.37]	<0.001
Alcohol			
Never	16926 (4.2%)	2010 (6.7%)	<0.001
Previous	12884 (3.2%)	2097 (7.0%)	
Current	373569 (92.3%)	25560 (85.7%)	
Education			
College or University degree	134781 (33.3%)	7362 (24.7%)	<0.001
A levels/AS levels or equivalent	45535 (11.3%)	2856 (9.6%)	
O levels/GCSEs or equivalent	84960 (21.0%)	6208 (20.8%)	
CSEs or equivalent	21770 (5.4%)	1715 (5.8%)	
NVQ or HND or HNC or equivalent	25931 (6.4%)	2080 (7.0%)	
Other professional qualifications	20280 (5.0%)	1689 (5.7%)	
None of the above	63677 (15.7%)	7217 (24.2%)	
ALS			
No	404216 (99.9%)	29767 (99.8%)	0.0106
Yes	415 (0.1%)	46 (0.2%)	

ALS, amyotrophic lateral sclerosis; BMI, body mass index; IMD, immune-mediated diseases.

*P<0.05, **P<0.01, ***P<0.001.

### 3.2 The association of any IMD and organ-specific IMDs with ALS incidence.

In our study, we found that a prior history of any IMD is associated higher risk of incident ALS when adjusting for age and sex in Model 1 (HR: 1.47, 95%CI: 1.08-1.99, **Table 2**). When further adjusting for socioeconomic indicators (education level and TDI) in Model 2, the results remained consistent (HR: 1.48, 95%CI: 1.09- 2.01, **Table 2**) with Model 1. Furthermore, when adjusting for lifestyle indicators (BMI, smoking, alcohol status) on the basis of Model 2, this result remained unchanged in Model 3(HR:1.42, 95%CI: 1.03-1.94, **Table 2**).

**Table 2:** Multivariate Cox analyses of any_IMD and organ_specific_IMDs associated with ALS incidence.

	Organ_specific_IMD	Model 1		Model 2		Model 3	
		HR (95% CI)	*P-value*	HR (95% CI)	*P-value*	HR (95% CI)	*P-value*
Any_IMD		1.47(1.08-1.99)	0.014^*^	1.48(1.09-2.01)	0.012^*^	1.42 (1.03-1.94)	0.032^*^
D_IMD	Diseases of the blood and blood-forming organs	1.61(0.23-11.46)	0.633	1.64(0.23-11.66)	0.622	1.70(0.24-12.12)	0.595
E_IMD	Endocrine, nutritional and metabolic diseases	3.20(1.66-6.19)	0.001^***^	3.21(1.66-6.22)	0.001^***^	3.01(1.49-6.06)	0.002^**^
G_IMD	Diseases of the nervous system	0.86(0.12-6.13)	0.881	0.88(0.12-6.25)	0.897	0.98(0.14-6.97)	0.984
I_IMD	Diseases of the circulatory system	1.02(0.14-7.24)	0.986	1.02(0.14-7.22)	0.988	1.03(0.15-7.35)	0.974
J_IMD	Diseases of the respiratory system	1.08(0.68-1.71)	0.739	1.09(0.69-1.72)	0.721	0.99(0.61-1.61)	0.975
K_IMD	Diseases of the digestive system	2.22(1.25-3.93)	0.006^**^	2.23(1.26-3.95)	0.006^**^	2.07(1.14-3.77)	0.017^*^
L_IMD	Diseases of the skin and subcutaneous tissue	1.49(0.48-4.64)	0.491	1.51(0.49-4.70)	0.475	1.57(0.50-4.88)	0.437
M_IMD	Diseases of the musculoskeletal system and connective tissue	1.25(0.56-2.80)	0.585	1.26(0.56-2.83)	0.569	1.30(0.58-2.92)	0.521

Model 1 adjusted for age, sex. Model 2 adjusted for age, sex, education, Townsend deprivation index. Model 3 adjusted for age, sex, education, Townsend deprivation index, BMI, smoking, alcohol consumption.

ALS, amyotrophic lateral sclerosis; BMI, body mass index; IMD, immune-mediated diseases; HR, hazard ratio; CI, confidence interval.

*P<0.05, **P<0.01, ***P<0.001.

When we classified IMDs based on eight different organ systems, we observed an elevated risk of developing ALS associated with two organ-specific IMDs. In all three models, IMDs of endocrine system were associated with increased risk of ALS (HR: 3.01, 95%CI: 1.49-6.06, **Table 2**), and IMDs of the digestive system were associated with increased risk of ALS (HR: 2.07, 95%CI: 1.14-3.77, **Table 2**).

### 3.3 Sensitivity and stratified analyses

Sensitivity analyses were performed to assess the robustness of the results. Participants with a short latency from initial sampling to diagnosis (1-year lag or 3-year lag) were excluded with the aim of excluding reverse causality, which did not substantially affect the results (**Table 3**, sensitivity analysis 1-2).

**Table 3:** Sensitive analysis of the peripheral immune markers on ALS incidence.

	Organ_specific_IMD	Sensitivity 1		Sensitivity 2	
		HR (95% CI)	*P-value*	HR (95% CI)	*P-value*
All_IMD		1.24(1.02-1.52)	0.034*	1.25(1.01-1.55)	0.040*
D_IMD	Diseases of the blood and blood-forming organs	1.75(0.25-12.49)	0.574	2.01 (0.28-14.29)	0.486
E_IMD	Endocrine, nutritional and metabolic diseases	3.11(1.54-6.27)	0.002**	2.70 (1.20-6.06)	0.016*
G_IMD	Diseases of the nervous system	1.01 (0.14-7.19)	0.992	0 (0.00-Inf)	0.985
I_IMD	Diseases of the circulatory system	1.07 (0.15-7.62)	0.946	1.24 (0.17-8.81)	0.831
J_IMD	Diseases of the respiratory system	1.02 (0.63-1.67)	0.923	0.96 (0.56-1.64)	0.887
K_IMD	Diseases of the digestive system	2.15 (1.18-3.90)	0.012*	2.01 (1.04-3.88)	0.039*
L_IMD	Diseases of the skin and subcutaneous tissue	1.62 (0.52-5.03)	0.407	0.62 (0.09-4.39)	0.629
M_IMD	Diseases of the musculoskeletal system and connective tissue	1.34 (0.60-3.01)	0.472	1.03 (0.38-2.76)	0.952

Sensitive analysis 1 was sensitive analysis of IMDs on ALS incidence with excluding ALS patients with a latency shorter than 1 year from initial sampling to diagnosis (1 year).

Sensitive analysis 2 was sensitive analysis of IMDs on ALS incidence with excluding ALS patients with a latency shorter than 3 years from initial sampling to diagnosis (3 years).

ALS, amyotrophic lateral sclerosis; BMI, body mass index; IMD, immune-mediated diseases; HR, hazard ratio; CI, confidence interval.

*P<0.05, **P<0.01, ***P<0.001.

The results of the stratified analysis indicate that the duration of IMDs has a significant impact on the development of ALS. Patients with IMD for over 10 years have higher risk of developing ALS (HR: 2.17, 95%CI: 1.12-4.21, **Table 4**), while a shorter duration (<10 years) does not affect ALS incidence. Additionally, the immune burden plays a crucial role in ALS incidence, as IMD with a heavy immune burden (CRP >= 1.3mg/L) is associated with an increased risk of ALS(HR:1.57, 95%CI: 1.09-2.28, **Table 4**), whereas IMD with a low immune burden does not influence ALS incidence(HR:1.07, 95%CI: 0.56-2.02, **Table 4**). When stratified by gender, we observed that female IMD patients are associated with an increased risk of ALS(HR:1.62, 95%CI: 1.06-2.48, **Table 4**), whereas male patients do not affect ALS incidence(HR:1.24, 95%CI:0.79-1.95, **Table 4**).

**Table 4:** Peripheral immunity and ALS risk across different IMD duration, CRP, and Sex subgroups.

		Any_IMD	
		HR (95% CI)	*P-value*
IMD Duration	<5y	1.50(0.99-2.26)	0.055
	>=5y, <10y	1.12(0.64-1.94)	0.699
	>=10y	2.17(1.12-4.21)	0.022 *
CRP	<1.3mmol/L	1.07 (0.56-2.02)	0.841
	>=1.3mmol/L	1.57(1.09-2.28)	0.016 *
Sex	female	1.62(1.06-2.48)	0.025 *
	male	1.24(0.79-1.95)	0.341

Model 1 adjusted for age, sex. Model 2 adjusted for age, sex, education, Townsend deprivation index. Model 3 adjusted for age, sex, education, Townsend deprivation index, BMI, smoking, alcohol consumption.

IMD, immune-mediated diseases; CRP, C-reactive protein; HR, hazard ratio; CI, confidence interval.

*P<0.05, **P<0.01, ***P<0.001.

## 4 Discussion

In the analyses from the large prospective cohort UK Biobank, we uncovered several key findings: 1) Immune-mediated diseases are significantly associated with an increased risk of incident ALS after adjusting for potential confounders; 2) Among these diseases, endocrine specific-IMDs and intestinal-specific IMDs were associated with higher risk of ALS incidence, while IMDs specific to the central nervous system are unrelated to the ALS risk. 3) Notably, such associations were effected by different immune burden reflected by the duration of IMDs and severity of inflammation. Longer duration of IMD (greater than 10 years) and higher CRP level (>1.3mg/L) were associated with a higher risk of ALS. Meanwhile, females with immune-mediated diseases were more prone to ALS than males.

To our knowledge, our study is the most comprehensive and largest cohort study to investigate the longitudinal association of IMDs on ALS risk. Our study suggested that previous IMD diagnosis was associated with higher risk of ALS incidence, while not simply exist together by chance. The coincidence of ALS and IMDs is likely due to shared pathological mechanisms and genetic susceptibility: 1) Patients with IMDs exhibit systemic immune dysregulation, including innate immune dysregulation (dysregulated immune cells, increased production of pro-inflammatory factors, complement activation) (28–31) and adaptive immune dysregulation (lymphocyte dysfunction) (32). Systematic inflammation may drive neuroinflammatory changes and chronic activation of microglia, leading to oxidative stress and deposition of misfolded proteins in ALS (33). 2) A genetic correlation study indicated potential genetic overlap between IMDs and ALS (19). Meanwhile, Hemminki et al. indicated that individuals with parents diagnosed with ALS had a higher risk of developing IMDs (34), suggesting a possible role of genetic factors in the IMD-ALS association. However, the results are not always consistent (21), more researches were needed to further demonstrate this association. These findings hold important clinical significance. Some ALS cases combined with IMDs have shown positive responses to immunotherapy, with symptoms reversing or stabilizing after immunotherapy (35, 36). Whether ALS patients with IMDs can benefit from immunotherapy should be assessed in clinical trials further. Early identification and intervention of IMDs may slow the onset of ALS, improve life quality and provide future care planning for ALS patients.

In our stratification analysis, our study newly found that heavier immune burden, including longer duration of IMDs and higher CRP levels were associated with higher risk of ALS. However, the positive association of longer duration of IMD with ALS incidence should be interpreted with caution, specially considering that the association with IMDs of less than 5 years shows almost a statistically significant result. This results was inconsistent with a previous study which found an increased in IMD just 2-5 years before ALS diagnosis but not before (21). This inconsistency may arise from that we only considered IMDs already present at baseline, which may have biased the results towards longer duration IMDs. We also found that females with IMDs were more prone to ALS than males. This sex-specific differences in IMD-ALS association may be due to the more active state of immune cells in females (37), which actively contribute to the differential incidences of ALS.

Specifically, we also found a very interesting association that intestinal-specific IMDs with higher risk of ALS incidence. Inflammatory bowel diseases (IBD), such as ulcerative colitis (UC), Crohn’s disease (CD), and celiac disease (CeD), are characterized by chronic, recurring inflammation localized in the intestines and can contribute to a systemic inflammatory burden (38). Over the past decade, several population-based cohorts have investigated the connections between IBD and other neurodegenerative diseases like Alzheimer’s (AD) and Parkinson’s (PD) (39–42). However, the relationship between IBD and ALS has been explored in limited observational cohorts. A case-control study from the United Kingdom found that the incidence of ALS in individuals with a history of UC was higher than in the healthy control group, although the difference was at a critical threshold (P ≈ 0.05), while CD had no impact on ALS incidence (20). In a nested case-control study from Sweden, no difference in the incidence of ALS was observed between those with UC, CD, and the control group (21). A genetic correlation study revealed a significant negative genetic correlation between UC, CD, and ALS, while a positive genetic correlation between CeD and ALS (19). Studies have also suggested a potential link between ALS and autoimmune reactivity and gluten sensitivity (43). In the study by Turner et al., individuals with a history of CeD had a 57% increased risk of developing ALS (20). However, these associations require further confirmation, as subsequent research has reported inconsistent results. A prospective cohort study from Sweden, which utilized small intestine biopsies for confirmation, did not find an association between pathologically confirmed CeD and ALS incidence (44). Taken together, these findings suggest a potential link between ALS and chronic gastrointestinal inflammation, but further research is needed. Several plausible mechanisms may contribute to increased ALS incidence in participants with IBD: 1) IBD is a chronic inflammatory disease involving not only intestinal but also extraintestinal inflammation, contributing to systemic inflammatory burden to individuals (45–47); 2) The exact cause of IBD is still unclear, but some theories suggest that the immune response of susceptible individuals to changes in the gut microbiota may be dysregulated. The gut-brain axis, describing the signaling between the gut, gut microbiota, and the central nervous system (CNS), is related to neurodegenerative changes. This bidirectional communication in the gut-brain axis is evident but also complex (48, 49). Research indicates that the gut microbiota has the ability to synthesize and release neurotransmitters and neuromodulators, such as short-chain fatty acids, dopamine, serotonin, and gamma-aminobutyric acid. The interaction between the gut and the brain occurs through the autonomic nervous system via the vagus nerve and the blood-brain barrier, both of which allow the passage of signaling molecules. Disruption of the intestinal epithelial barrier and dysbiosis of the microbiota caused by IBD may promote the entry of neurotoxic metabolites originating from the gut microbiota into the central nervous system (50, 51). A multicenter, randomized, double-blind clinical trial of therapeutic intervention involving fecal microbiota transplantation(FMT) in ALS patients (NCT03766321) aimed to illustrate that the re-establishment of an appropriate microenvironment can potentially disrupt the immune response and disease progression in ALS (52), but the results were not published. According to existing clinical evidence, FMT exhibits a reasonable cure rate and carries a low incidence of severe adverse events, and being well-tolerated (53), which is a promising therapeutic method for ALS coexisting IMDs.

Our findings also revealed a positive association between endocrine-specific IMDs [type 1 diabetes(T1D), Graves’ disease and autoimmune thyroiditis], with the risk of ALS incidence. Previous retrospective study found an elevated ALS incidence among individuals with a history of T1D in those with lower age (20). This intriguing finding aligns with the conclusions of our research, although we did not further categorize endocrine-specific IMDs further, given the relatively small number of ALS cases. Notably, T1D is more frequently associated with weight loss, which is linked to faster ALS progression (54). As for studies regarding thyroid IMDs and ALS incidence, Cui et al. found a positive association between ALS and autoimmune hypothyreosis (21), which is also aligns with our conclusion. Given that ALS primarily pertains to motor neurons, the endocrine system is not a primary participant in its pathophysiology. The association between the endocrine system and ALS has yet to be clearly established. Since ALS is a progressively fatal disease, addressing potential endocrine effects in ALS patients is crucial for reducing the overall burden of ALS-related conditions.

Our study has several strengths: 1) To our knowledge, our study is the first prospective cohort examining the relationship between immune-mediated diseases and the incidence of ALS, with a large cohort size; 2) We investigated the immune burden, including the duration of IMDs and severity of inflammation, for the first time on ALS onset; 3) Our study also made novel findings regarding organ-specific IMDs, especially the intestinal and endocrine-specific IMDs on ALS risk. However, our study also has several limitations:1) Despite being a prospective cohort, causation should be interpreted with caution; 2) Due to the rarity of ALS, the incidence of ALS is low in some rare IMDs, which could lead to biased results. Therefore, we did not further differentiate the impact of individual IMD on ALS; 3) Data on central immune cells such as microglia, humoral immune biomarkers like interleukins and protein aggregates such as TDP43 are not available in the UK Biobank. 4) Some immune diseases may have symptoms similar to ALS, such as neurological symptoms caused by CeD, which could lead to less accurate data; 5) Medication information was not available. 6) It is not possible to precisely define IMDs and we can only refer to the previous studies. The potential selection bias is ineluctable. 7) The baseline characteristics between people with and without IMDs were imbalanced, although we performed multivariable models adjusting for those baseline characteristics to mitigate the imbalance.

In conclusion, the increased risk of ALS in patients with IMDs and the findings from stratified analysis suggest that this population may benefit from a multidisciplinary approach to enhance clinical awareness. Future research directions include a deeper exploration of the biological connections between IMDs and ALS, as well as the search for potential therapies to enhance the quality of life and prognosis for ALS patients. This may involve novel approaches such as manipulating the gut-brain axis through microbiota manipulation or hormone therapy. Research in this field holds the promise of providing further insights into the etiology and progression of ALS, advancing our understanding of this disease, and opening up new avenues for future treatment strategies.

## Data availability statement

The datasets presented in this study can be found in online repositories. The names of the repository/repositories and accession number(s) can be found below: This study was conducted using UK Biobank resource (application number, 79095).

## Ethics statement

The studies involving humans were approved by the UK National Health Service, National Research Ethics Service North West, the National Information Governance Board for Health and Social Care in England and Wales, and the Community Health Index Advisory Group in Scotland. The studies were conducted in accordance with the local legislation and institutional requirements. Written informed consent for participation was not required from the participants or the participants’ legal guardians/next of kin in accordance with the national legislation and institutional requirements.

## Author contributions

WC: Conceptualization, Data curation, Formal Analysis, Investigation, Methodology, Software, Writing – original draft, Writing – review & editing. ZC: Writing – original draft, Writing – review & editing, Conceptualization, Data curation, Formal Analysis, Investigation, Methodology. LT: Validation, Visualization, Writing – review & editing. CX: Formal Analysis, Project administration, Writing – original draft, Writing – review & editing. DF: Writing – original draft, Writing – review & editing, Funding acquisition, Validation.
