# Supplementary material for: Immune-mediated diseases are associated with a higher risk of ALS incidence: a prospective cohort study from the UK Biobank
Source: Front Immunol. 2024 Mar 5;15:1356132. doi: 10.3389/fimmu.2024.1356132 (PMC10948436; doi:10.3389/fimmu.2024.1356132)
Supplement: Supplementary file 1 [file Table_1.docx]

Supplementary materials

Table 1 The full results of model 3 after removed all IMD participants after baseline.

| **Exposures** | **HR** | **95%CI** | **P** |
| --- | --- | --- | --- |
| Age | 1.087 | 1.072 - 1.103 | 0***** |
| Sex | 1.521 | 1.258 - 1.84 | 0***** |
| Education | 0.993 | 0.971 - 1.016 | 0.559 |
| Smoking | 1.074 | 0.937 - 1.231 | 0.304 |
| Alcohol | 0.923 | 0.765 - 1.113 | 0.401 |
| TDI | 0.994 | 0.963 - 1.026 | 0.715 |
| BMI | 0.995 | 0.974 - 1.016 | 0.617 |
| **all_IMD** | 1.416 | 1.031 - 1.944 | 0.032*** |

*Abbreviations: TDI: Townsend deprivation index; BMI: body mass index; IMD: immune-mediated diseases. HR: hazard ratio; CI: confidence interval;*

**P<0.05, **P<0.01, ***P<0.001.*

Table 2 The full results of model 3 after removed all D_IMD participants after baseline.

| **Exposures** | **HR** | **95%CI** | **P** |
| --- | --- | --- | --- |
| **Age** | 1.083 | 1.069 - 1.097 | 0***** |
| **Sex** | 1.421 | 1.196 - 1.69 | 0***** |
| **Education** | 0.988 | 0.968 - 1.009 | 0.259 |
| **Smoking** | 1.048 | 0.926 - 1.187 | 0.456 |
| **Alcohol** | 0.972 | 0.813 - 1.161 | 0.751 |
| **TDI** | 0.997 | 0.969 - 1.027 | 0.865 |
| **BMI** | 0.994 | 0.976 - 1.013 | 0.561 |
| **D_IMD** | 1.703 | 0.239 - 12.119 | 0.595 |

*Abbreviations: TDI: Townsend deprivation index; BMI: body mass index; IMD: immune-mediated diseases. HR: hazard ratio; CI: confidence interval;*

**P<0.05, **P<0.01, ***P<0.001.*

Table 3 The full results of model 3 after removed all E_IMD participants after baseline.

| **Exposures** | **HR** | **95%CI** | **P** |
| --- | --- | --- | --- |
| **Age** | 1.082 | 1.068 - 1.097 | 0***** |
| **Sex** | 1.416 | 1.191 - 1.683 | 0***** |
| **Education** | 0.99 | 0.97 - 1.01 | 0.316 |
| **Smoking** | 1.048 | 0.926 - 1.187 | 0.459 |
| **Alcohol** | 0.965 | 0.809 - 1.152 | 0.697 |
| **TDI** | 0.998 | 0.969 - 1.027 | 0.892 |
| **BMI** | 0.993 | 0.974 - 1.012 | 0.453 |
| **E_IMD** | 3.008 | 1.493 - 6.06 | 0.002**** |

*Abbreviations: TDI: Townsend deprivation index; BMI: body mass index; IMD: immune-mediated diseases. HR: hazard ratio; CI: confidence interval;*

**P<0.05, **P<0.01, ***P<0.001.*

Table 4 The full results of model 3 after removed all G_IMD participants after baseline.

| **Exposures** | **HR** | **95%CI** | **P** |
| --- | --- | --- | --- |
| **Age** | 1.084 | 1.07 - 1.098 | 0***** |
| **Sex** | 1.415 | 1.188 - 1.685 | 0***** |
| **Education** | 0.99 | 0.969 - 1.01 | 0.316 |
| **Smoking** | 1.058 | 0.934 - 1.2 | 0.376 |
| **Alcohol** | 0.953 | 0.799 - 1.137 | 0.593 |
| **TDI** | 0.998 | 0.97 - 1.028 | 0.918 |
| **BMI** | 0.993 | 0.974 - 1.012 | 0.468 |
| **G_IMD** | 0.98 | 0.138 - 6.971 | 0.984 |

*Abbreviations: TDI: Townsend deprivation index; BMI: body mass index; IMD: immune-mediated diseases. HR: hazard ratio; CI: confidence interval;*

**P<0.05, **P<0.01, ***P<0.001.*

Table 5 The full results of model 3 after removed all I_IMD participants after baseline.

| **Exposures** | **HR** | **95%CI** | **P** |
| --- | --- | --- | --- |
| **Age** | 1.084 | 1.07 - 1.098 | 0***** |
| **Sex** | 1.447 | 1.215 - 1.722 | 0***** |
| **Education** | 0.988 | 0.967 - 1.008 | 0.231 |
| **Smoking** | 1.064 | 0.939 - 1.206 | 0.332 |
| **Alcohol** | 0.982 | 0.818 - 1.178 | 0.842 |
| **TDI** | 1.001 | 0.972 - 1.031 | 0.931 |
| **BMI** | 0.995 | 0.976 - 1.014 | 0.609 |
| **I_IMD** | 1.033 | 0.145 - 7.351 | 0.974 |

*Abbreviations: TDI: Townsend deprivation index; BMI: body mass index; IMD: immune-mediated diseases. HR: hazard ratio; CI: confidence interval;*

**P<0.05, **P<0.01, ***P<0.001.*

Table 6 The full results of model 3 after removed all J_IMD participants after baseline.

| **Exposures** | **HR** | **95%CI** | **P** |
| --- | --- | --- | --- |
| **Age** | 1.083 | 1.069 - 1.098 | 0***** |
| **Sex** | 1.453 | 1.213 - 1.739 | 0***** |
| **Education** | 0.992 | 0.971 - 1.013 | 0.432 |
| **Smoking** | 1.043 | 0.917 - 1.187 | 0.519 |
| **Alcohol** | 0.938 | 0.784 - 1.123 | 0.488 |
| **TDI** | 0.997 | 0.967 - 1.027 | 0.821 |
| **BMI** | 0.999 | 0.98 - 1.019 | 0.946 |
| **J_IMD** | 0.992 | 0.611 - 1.613 | 0.975 |

*Abbreviations: TDI: Townsend deprivation index; BMI: body mass index; IMD: immune-mediated diseases. HR: hazard ratio; CI: confidence interval;*

**P<0.05, **P<0.01, ***P<0.001.*

Table 7 The full results of model 3 after removed all K_IMD participants after baseline.

| **Exposures** | **HR** | **95%CI** | **P** |
| --- | --- | --- | --- |
| **Age** | 1.084 | 1.07 - 1.098 | 0***** |
| **Sex** | 1.431 | 1.202 - 1.704 | 0***** |
| **Education** | 0.988 | 0.968 - 1.008 | 0.234 |
| **Smoking** | 1.043 | 0.921 - 1.183 | 0.506 |
| **Alcohol** | 0.955 | 0.801 - 1.14 | 0.611 |
| **TDI** | 0.996 | 0.967 - 1.026 | 0.784 |
| **BMI** | 0.994 | 0.975 - 1.013 | 0.528 |
| **K_IMD** | 2.072 | 1.14 - 3.766 | 0.017*** |

*Abbreviations: TDI: Townsend deprivation index; BMI: body mass index; IMD: immune-mediated diseases. HR: hazard ratio; CI: confidence interval;*

**P<0.05, **P<0.01, ***P<0.001.*

Table 8 The full results of model 3 after removed all L_IMD participants after baseline.

| **Exposures** | **HR** | **95%CI** | **P** |
| --- | --- | --- | --- |
| **Age** | 1.082 | 1.068 - 1.096 | 0***** |
| **Sex** | 1.411 | 1.186 - 1.679 | 0***** |
| **Education** | 0.99 | 0.97 - 1.01 | 0.323 |
| **Smoking** | 1.043 | 0.92 - 1.182 | 0.514 |
| **Alcohol** | 0.971 | 0.812 - 1.162 | 0.75 |
| **TDI** | 0.995 | 0.966 - 1.025 | 0.749 |
| **BMI** | 0.994 | 0.975 - 1.014 | 0.562 |
| **L_IMD** | 1.569 | 0.504 - 4.883 | 0.437 |

*Abbreviations: TDI: Townsend deprivation index; BMI: body mass index; IMD: immune-mediated diseases. HR: hazard ratio; CI: confidence interval;*

**P<0.05, **P<0.01, ***P<0.001.*

Table 9 The full results of model 3 after removed all M_IMD participants after baseline.

| **Exposures** | **HR** | **95%CI** | **P** |
| --- | --- | --- | --- |
| **Age** | 1.083 | 1.068 - 1.097 | 0***** |
| **Sex** | 1.432 | 1.199 - 1.711 | 0***** |
| **Education** | 0.991 | 0.971 - 1.012 | 0.414 |
| **Smoking** | 1.059 | 0.932 - 1.204 | 0.376 |
| **Alcohol** | 0.959 | 0.799 - 1.152 | 0.658 |
| **TDI** | 0.991 | 0.962 - 1.022 | 0.573 |
| **BMI** | 0.991 | 0.971 - 1.01 | 0.35 |
| **M_IMD** | 1.302 | 0.582 - 2.917 | 0.521 |

*Abbreviations: TDI: Townsend deprivation index; BMI: body mass index; IMD: immune-mediated diseases. HR: hazard ratio; CI: confidence interval;*

**P<0.05, **P<0.01, ***P<0.001.*
